# Goal-directed therapy in patients with early acute kidney injury: a multicenter randomized controlled trial

**DOI:** 10.6061/clinics/2018/e327

**Published:** 2018-10-25

**Authors:** Cristina Prata Amendola, João Manoel Silva-Jr, Taisa Carvalho, Luciana Coelho Sanches, Ulysses Vasconcelos de Andrade e Silva, Rosana Almeida, Emmanuel Burdmann, Emerson Lima, Fabiana Ferreira Barbosa, Renata Souza Ferreira, Maria José C Carmona, Luiz Marcelo Sá Malbouisson, Fernando A M Nogueira, José Otavio Costa Auler-Júnior, Suzana Margareth Lobo

**Affiliations:** I Barretos Cancer Hospital, Barretos, SP, BR; II Instituto de Assistencia Medica ao Servidor Publico Estadual, Hospital do Servidor Publico Estadual (HSPE), Sao Paulo, SP, BR; III Divisao de Nefrologia, Faculdade de Medicina FMUSP, Universidade de Sao Paulo, Sao Paulo, SP, BR; IV Faculdade de Medicina de Sao Jose do Rio Preto, Sao Jose do Rio Preto, SP, BR; V Divisao de Anestesiologia e Terapia Intensiva Cirurgica, Instituto do Coracao (InCor), Divisao de Anestesiologia do Hospital das Clinicas HCFMUSP, Faculdade de Medicina, Universidade de Sao Paulo, Sao Paulo, SP, BR

**Keywords:** Oxygen Consumption, Hemodynamics, Acute Kidney Injury, Creatinine, Perfusion

## Abstract

**OBJECTIVES:**

Acute kidney injury is associated with many conditions, and no interventions to improve the outcomes of established acute kidney injury have been developed. We performed this study to determine whether goal-directed therapy conducted during the early stages of acute kidney injury could change the course of the disease.

**METHODS:**

This was a multicenter prospective randomized controlled study. Patients with early acute kidney injury in the critical care unit were randomly allocated to a standard care (control) group or a goal-directed therapy group with 8h of intensive treatment to maximize oxygen delivery, and all patients were evaluated during a period of 72h. ClinicalTrials.gov: NCT02414906.

**RESULTS:**

A total of 143 patients were eligible for the study, and 99 patients were randomized. Central venous oxygen saturation was significantly increased and the serum lactate level significantly was decreased from baseline levels in the goal-directed therapy group (*p*=0.001) compared to the control group (*p*=0.572). No significant differences in the change in serum creatinine level (*p*=0.96), persistence of acute kidney injury beyond 72h (*p*=0.064) or the need for renal replacement therapy (*p*=0.82) were observed between the two groups. In-hospital mortality was significantly lower in the goal-directed therapy group than in the control group (33% *vs*. 51%; RR: 0.61, 95% CI: 0.37-1.00, *p*=0.048, number needed to treat=5).

**CONCLUSIONS:**

Goal-directed therapy for patients in the early stages of acute kidney injury did not change the disease course.

## INTRODUCTION

Acute kidney injury (AKI) is currently defined by abrupt and small changes in serum creatinine (SCr) levels, which are associated with adverse short-term and long-term outcomes. Even transient episodes of oliguria appear to be associated with long-term hazards 1, 2. The estimated overall incidence of AKI in the intensive care unit (ICU) ranges from 11% to 100%, and its mortality may be greater than 68% in septic patients 3, 4. More than 50% of the patients at risk for AKI develop kidney injury or failure 5. Diuretics, low doses of dopamine, fenoldopam, atrial natriuretic peptide, erythropoietin, and growth hormones have all failed as preventive or treatment measures in AKI patients 3,6-12.

In tertiary hospitals, the leading clinical conditions associated with AKI are major surgery, hypovolemia, heart failure and sepsis, which share an underlying hypoperfusion pathogenesis. In fact, ischemia, alone or associated with nephrotoxic drugs, may be considered the main etiological cause of in-hospital and ICU AKI 13. However, the interplay of other complex mechanisms, such as adaptation responses, inflammation, microvascular dysfunction, metabolic downregulation and reprioritization of energy, also contribute to the development of AKI 13,14.

The correction of volume deficits and optimization of hemodynamic status can minimize kidney injury and potentially facilitate recovery from AKI in critically ill patients. Several studies suggest that a protocol-based management of hemodynamic and oxygenation parameters, which is known as goal-directed therapy (GDT), can prevent the development or worsening of AKI 14-17. The goal of this approach is to maximize oxygen delivery (DO_2_) and cardiac output (CO) or to at least prevent tissue hypoxia due to an imbalance between DO_2_ and oxygen consumption (VO_2_) 18. Furthermore, GDT has been shown to significantly decrease the complications and risk of death in high-risk patients undergoing noncardiac surgery 19. The Kidney Disease: Improving Global Outcomes (KDIGO) Clinical Practice Guideline suggests using protocol-based management of hemodynamic and oxygenation parameters to prevent the development or worsening of AKI in high-risk patients in the perioperative setting or in patients with septic shock 20.

A meta-analysis of the effects of GDT on postoperative renal dysfunction reported that surgical patients receiving perioperative hemodynamic optimization, particularly with fluids and inotropic drugs, have a significantly lower risk of renal impairment 14. Postoperative GDT with fluid challenges aimed at increasing the stroke volume by at least 10% after cardiac surgery was associated with reductions in the incidence of AKI, ICU length of stay (LOS), and hospital LOS 17. The use of a modified goal-directed protocol resulted in a faster recovery from septic shock and a lower risk of developing renal failure 15. Patients with sepsis have a very high incidence of AKI, and although an early GDT protocol showed no specific benefits on AKI outcomes, it did have an impact on other organ failures 16. Moreover, GDT improved long-term outcomes in noncardiac surgical patients during a 15-year follow-up study 19. Thus, the maintenance of adequate renal blood flow is the primary strategy to assure renal oxygenation and to prevent AKI. Therefore, we hypothesize that optimization of the hemodynamic status and correction of volume deficit during the early stages of AKI will help minimize further extension of kidney injury and will facilitate recovery from AKI.

## MATERIALS AND METHODS

This multicenter prospective randomized controlled study was approved by the Institutional Review Board of each of the following institutions: CEP Hospital do Câncer de Barretos, CEP Hospital do Servidor Público Estadual, CEP Hospital das Clinicas - FMUSP, and CEP Faculdade de Medicina de São José do Rio Preto - FAMERP, in accordance with the ethical standards of the responsible committee on human experimentation and the Helsinki Declaration. The study was conducted at one surgical ICU, one oncological ICU and two mixed ICUs from four tertiary hospitals in Brazil from 2011 to 2014 (Barretos Cancer Hospital - Fundação PIO XII, Servidor Público Estadual Hospital - IAMSP, Hospital das Clínicas of the University of São Paulo, and Hospital de Base de São José do Rio Preto of the Sao Jose do Rio Preto Medical School). Informed consent was obtained from all patients or their next of kin. The protocol was registered at clinicaltrials.gov (NCT02414906).

**Figure 1:**
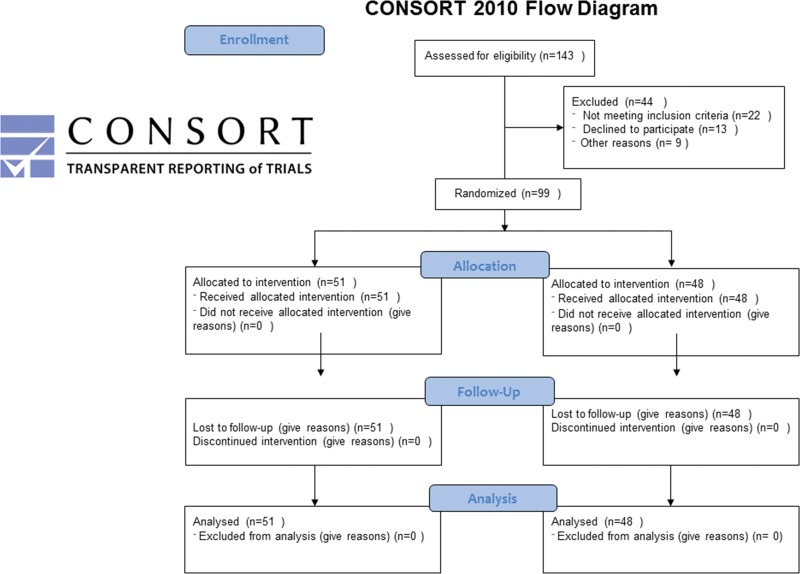
Randomization and follow-up of patients in the control and GDT groups.

Patients were eligible if they were in an early stage of AKI, defined as an increase in SCr≥0.3 mg/dL over no more than 12h and/or a urine output of less than 0.5 mL/kg/h over 6h and for less than 12h that developed during the first nine days of ICU admission. The exclusion criteria were age <18 years, chronic kidney disease requiring hemodialysis, nephrectomy, SCr≥4 mg/dL at any time after hospital admission, life expectancy <90 days, pregnancy, severe cardiac arrhythmias (receiving drug therapy), severe heart failure classified as New York Heart Association III or IV (NYHA III or IV), or acute myocardial infarction (AMI) (Figure 1).

The patients were assigned to either the GDT group or the conventional therapy group (control). Patients were randomly allocated using numbered, opaque, sealed envelopes containing computer-generated random allocations in a ratio of 1:1 and in blocks of ten. Demographic and clinical data were obtained, and patients with histories of diabetes mellitus, autoimmune disease, and use of nephrotoxic drugs were registered. Severe sepsis and septic shock were defined according to the American College of Chest Physicians/Society of Critical Care Medicine (ACCP/SCCM) guidelines 20. Acute Physiology and Chronic Health Evaluation Classification System II (APACHE II) and Sequential Organ Failure Assessment (SOFA) scores were determined after enrollment in the study 21,22. The SOFA score was determined again after 24h (SOFA 24h), and the change in the SOFA score was calculated.

Electrocardiography (EKG), heart rate (HR), peripheral blood oxygen saturation (SaO2), central venous pressure (CVP), urinary output, and mean arterial pressure (MAP) were monitored during the study period and registered every 2h over an 8-h period in both groups. Hemoglobin (Hb), arterial blood gases, and serum lactate levels were obtained at baseline (before GDT) and at 4h and 8h after initiation of GDT. SCr levels were assessed at baseline and daily for 3 days. SCr differences were calculated as the difference between these measurements and the baseline measurement.

The control group was treated at the discretion of physicians who were not involved in the current study according to physiological parameters, such as urinary output, HR, and MAP, if necessary. Serum lactate, central venous oxygen saturation (ScvO_2_) (if a central venous catheter was available), and Hb values were collected by study coordinators at the beginning of the study period, at 4h after the beginning, and at the end of the 8-h study period.

**Figure 2:**
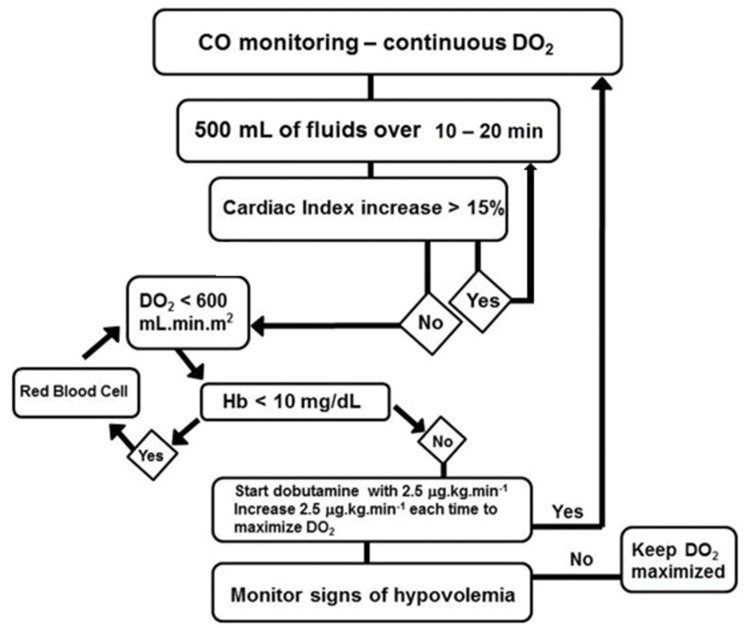
Goal-direct therapy (GDT) algorithm used in this study.

In the GDT group, a minimally invasive technique utilizing the FlowTrac system and the Vigileo monitor (Edwards Lifesciences; Irvine, CA, USA) was applied to continuously monitor the cardiac output (CO), oxygen delivery index (IDO_2_), and systemic vascular resistance index (SVRI). The CO, IDO_2_, SVRI, and ScvO_2_ were monitored at baseline and every 2h for 8h. The study investigators had to be present for the randomization and initiation of the study procedures. The treatment algorithm was performed by one of the study investigators (Figure 2). The goal was to maximize IDO_2_ using fluid challenges, red blood cell infusion if the Hb level was <10 mg/dL, and dobutamine, beginning at 2.5 μg/kg/min and progressively increasing until the goal (to maintain IDO_2_ as close to 600 mL/min/m^2^ as possible) was reached during the 8-h study period. The fluid challenge used to assess the CO response involved infusion of 500 mL of 0.9% saline solution every 30 min. A patient was considered a fluid-responder when a stable 15% increase in the CO was observed. Dobutamine administration was interrupted in cases of predefined adverse events (persistent tachycardia, hypotension unresponsive to fluid challenge, angina, and/or signs of myocardial ischemia on EKG). Noradrenaline was used in the presence of hypotension (MAP <65 mmHg), and sodium nitroprusside was used if the MAP was higher than 90 mmHg. Patients in the two groups were followed until hospital discharge or death. The ICU LOS, hospital LOS, and need for renal replacement therapy (RRT) were assessed.

### Study outcomes

The primary objective of the present study was to determine whether GDT during the early stage of AKI (AKI Network stage 1 or AKIN 1) could decrease the risk of renal impairment due to the persistence of impaired renal function longer than 72h (SCr level after 72h higher than before GDT). The secondary objective was to assess the impact of GDT on tissue perfusion, ICU LOS, hospital LOS, in-hospital mortality and the composite of hospital death, RRT, and the persistence of impaired renal function after 72h.

### Statistical analysis

The sample size was determined based on the probability that an ICU patient in an early phase of renal injury (stage 1) would progress to more severe stages of AKI during the ICU stay; this probability was estimated to be 60% for the control group 4. The effect size was estimated based on studies in which perioperative GDT decreased the incidence of AKI by more than 50% 14. To achieve a study power of 80% and a significance level of 0.05 using a two-sided test assuming a 50% decrease in AKI incidence, 48 patients were required for each group. Continuous variables are presented as the mean ± standard deviation or the median with interquartile range, and categorical variables are given as the number and percentage, unless otherwise indicated. The Kolmogorov-Smirnov test was used to verify the normality of the distribution of quantitative variables.

Difference testing between groups was performed using a two-tailed *t-*test, Chi-Square, Fisher, or Mann-Whitney test as appropriate. The Friedman test was used to compare repeated measurements of nonnormally distributed variables. When statistically significant differences were observed, the Bonferroni correction was used to detect the time points at which the differences were significant. The incidences of complications and mortality were evaluated using the relative risk (RR) [95% confidence interval (CI)]. Two-tailed *p-*values <0.05 indicated statistically significant results.

## RESULTS

A total of 143 patients were eligible during the enrollment period from April 2010 to December 2013, of which 44 were excluded due to the following: lack of informed consent (n=13), monitoring tools not available (n=9), AKI longer than 12h (n=7), cardiac arrhythmia (n=7), recent myocardial infarction (n=3), nephrectomy (n=2), ICU LOS longer than 10 days (n=1) and survival expectancy less than 3 months (n=2). The cumulative percentages of patients included were as follows: day 0: 22%, day 1: 73%, day 2: 86%, day 3: 89%, day 4: 94%, day 5: 97%, day 6: 98% and day 8: 100%.

The mean age of the patients was 65.7±13.9 years, and the median APACHE II and SOFA scores were 16 [11.3-21.0] and 4.0 [2.0-7.0], respectively. Eighty-seven percent of the total patient population consisted of surgical patients, of whom 32% were submitted to emergency surgery, 32% had sepsis, 55% had cancer, and 55% underwent gastrointestinal surgeries.

The baseline characteristics of the two groups are shown in Table 1. The groups did not differ significantly in age, APACHE II score, SOFA score or baseline SCr. Table 2 shows the interventions in the 8-h study period for both groups. More patients in the GDT group received fluid challenges than those in the control group (92% *vs*. 57%, *p*<0.001). The median number of fluid challenges was also greater in the GDT group (3 *vs*. 2, *p*=0.008). During the 8-h study period, the GDT group received a nonsignificantly larger volume of crystalloids (*p*=0.318).

More patients in the GDT group received red blood cell transfusions (54% *vs*. 4%, *p*<0.001), dobutamine (79% *vs*. 8%, *p*=0.001), and sodium nitroprusside (12.0% *vs*. 2.0%, *p*=0.054) than those in the control group. However, colloids (24% *vs*. 0%, *p*<0.001) and furosemide (16% *vs*. 2%, *p*=0.031) were more frequently used in the control group (Table 2).

**Figure 3:**
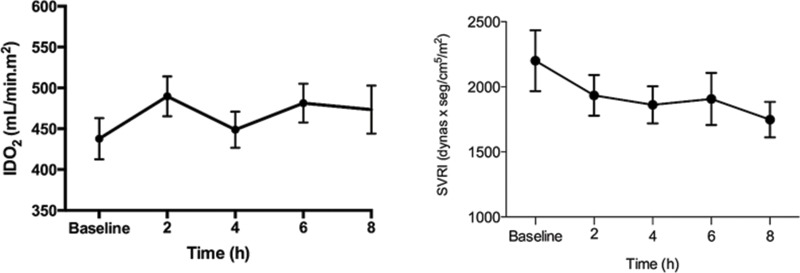
Oxygen delivery index (IDO2) and systemic vascular resistance index (SVRI) in the GDT and control groups during the 8-h study period.

Vital signs, CVP, Hb, peripheral blood oxygen saturation (SaO_2_), and perfusion variables during the 8-h study period are shown in Table 3. The HR was significantly increased from baseline in both groups. No difference in the MAPs of the two groups were observed at any time. The CVP of the GDT group was lower than that of the control group at baseline and at 6h (baseline: 9 *vs*. 12 mmHg, *p*=0.02; 6h: 10 *vs*. 13 mmHg, *p*=0.04). Only the GDT group exhibited a significant increase in CVP during treatment. Moreover, the IDO_2_ was significantly increased in the GDT group [from 409 mL/min.m^2^ at baseline to 436 mL/min.m^2^ at 2h (*p*=0.001) and 461 mL/min.m^2^ at 6h (*p*=0.015)] (Figure 3), while the SVRI was decreased in the GDT group (Figure 3). However, only 18 patients (37%) achieved IDO_2_ values higher than 600 mL/min.m^2^.

**Figure 4:**
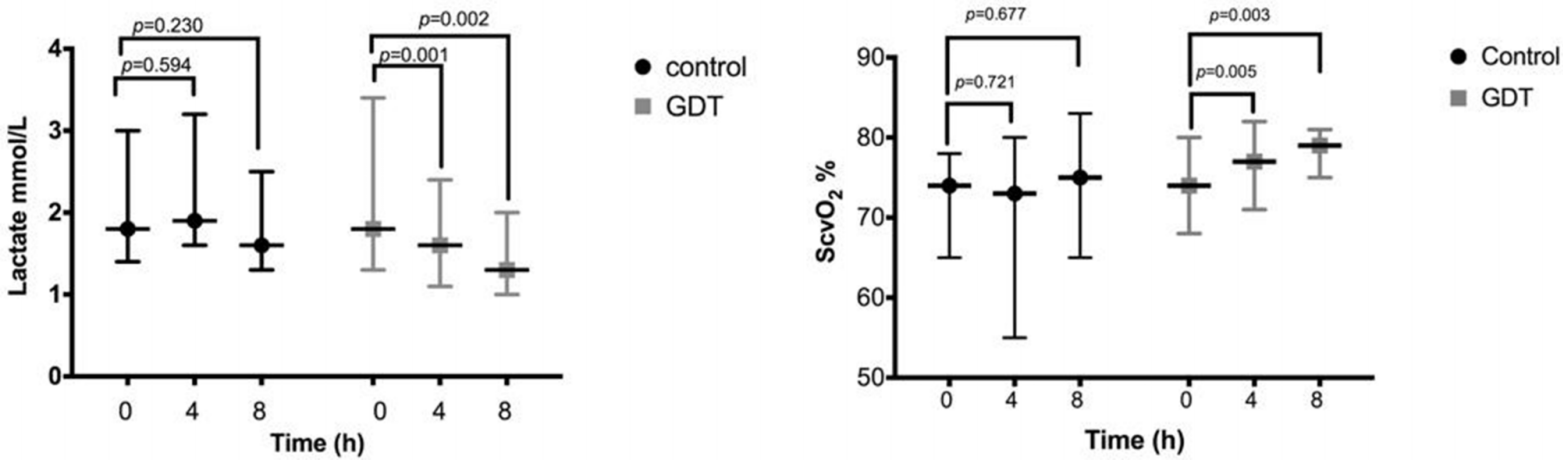
Central venous oxygen saturation and serum lactate levels in the GDT and control groups during the 8-h study period.

The ScvO_2_ in the GDT group was significantly higher at 4h (*p*=0.005) and 8h (*p*=0.003) than at baseline and significantly higher than that in the control group at 8h (79% *vs*. 75%, *p*=0.03) (Figure 4). The serum lactate concentration did not differ significantly between the GDT and control groups at baseline (*p*=0.75), 4h (*p*=0.16), or 8h (*p*=0.20). However, the serum lactate level in the GDT group significantly declined during the 4-h and 8-h periods (*p*=0.001 and *p*=0.002) (Figure 4).

No significant differences were found in the SCr level or the need for RRT between the two groups (*p*=0.82) (Table 4). No other parameter of renal function, such as diuresis at 8h (398 mL *vs*. 397 mL; *p*=0.515), differed significantly between the GDT and control groups (Table 4). The prevalence of AKI persistence longer than 72h was 78.4% in the control group *vs*. 62.5% in the GDT group (RR: 0.79; 95% CI: 0.61-1.03; *p*=0.064; number needed to treat=6) (Table 4).

No significant effect of GDT was observed on ICU LOS (*p*=0.58), hospital LOS (*p*=0.74), or ICU mortality (*p*=0.33); however, in-hospital mortality was significantly decreased in the GDT group (33% *vs*. 51%; RR: 0.61, 95% CI: 0.37-1.00; *p*=0.048; number needed to treat=5) (Table 4). The composite outcome was not significantly decreased in the GDT group (77.1% *vs*. 86.3%; RR: 0.89; 95% CI: 0.74-1.07; *p*=0.18; number needed to treat=11).

## DISCUSSION

This is the first study on the clinical benefits of DO_2_ optimization using GDT during the early phase of AKI (AKIN 1). The main findings of our study are that GDT prevented tissue hypoxia and decreased mortality relative to standard therapy but did not change the course of AKI. These results suggest that an elevation in SCr might be a late marker for AKI development, an early marker for a higher mortality risk and a possible identifier of patients who are likely to benefit from GDT.

GDT has been recommended for preventing the development or worsening of AKI in high-risk patients in the perioperative setting 23. In a meta-analysis of 20 randomized controlled trials, Brienza et al. 14 concluded that postoperative AKI was reduced by 56% and that mortality was reduced by 50% compared to the control treatment by perioperative hemodynamic optimization. However, hemodynamic optimization was preemptive in the studies included, while our study population was a heterogeneous patient group admitted to the ICU, following surgery for most (87%); thus, GDT was not preemptive. Our results suggest that when the SCr level increases, kidney injury is already established, and GDT cannot change its course, possibly because AKI is no longer volume-responsive or blood flow-responsive. Nevertheless, our results suggest that early increases in SCr may be an early marker of severity and that more aggressive supportive measures, as performed in the GDT group, should be offered to high-risk patients.

Serum lactate and ScvO_2_ levels have previously been used as markers of hypoperfusion and as guides for the resuscitation of critically ill patients 24. Indeed, lactate elevation parallels that of mortality, and even a mild elevation in lactate is a risk factor for worse outcomes 24. The greater decrease in serum lactate and better recovery of ScvO_2_ in the GDT group suggest that the presence of global occult hypoperfusion is still responsive to an enhanced DO_2_ in early-stage AKI patients. Jansen et al. 25 reported that lactate monitoring and treatment directed at decreasing lactate levels in ICU patients with a serum lactate level ≥3.0 mEq/L significantly decreased mortality. In septic shock patients, Washarasint et al. reported that higher serum lactate levels, even those within a normal range, were associated with higher mortality rates in septic patients 26. The mortality in septic patients with serum baseline lactate levels between 1.4 and 2.3 mEq/L was almost doubled compared to those with levels lower than 1.4 mEq/L, which stresses that even lower values within the threshold range may require clinical attention 26. In another study, an analysis of lactate clearance cut-off values showed that a value of <10% exhibited the maximum sum of sensitivity plus specificity for predicting in-hospital mortality, and the high-clearance group had a 52% relatively lower in-hospital mortality rate than the low-clearance group 27. Accordingly, along with inducing faster clearance of serum lactate and recovery of ScvO_2_, GDT was associated with significantly decreased in-hospital mortality.

The main strength of our study is its multicenter, prospective, controlled, and randomized design. The main limitation of this study is the possibility of a type II error due to its small sample size. Nonetheless, the high *p*-values for the primary outcomes (the need for RRT, *p*=0.82 and the SCr difference on day 3 compared to baseline, *p*=0.914) strongly suggest that no differences would be present between the two groups (i.e., regarding the benefit of GDT) even with a larger sample size. Another possible issue with our study is that the time window of 12h that was allowed between the diagnosis of AKI and the start of GDT may have resulted in irreversible kidney damage, thereby underestimating the therapeutic advantage of GDT compared to that of standard treatment. Perhaps earlier initiation of GDT could yield a greater recovery of renal function. However, the median time to GDT initiation in this study (6h) was required to obtain informed consent and conduct baseline monitoring. More likely, renal dysfunction is a result of an interplay of more complex mechanisms, such as adaptive responses, inflammation, microvascular dysfunction and metabolic and bioenergetic downregulation, that cannot be modified by GDT 13. Finally, it is possible that novel AKI biomarkers other than SCr may better screen for patients who are likely to benefit from GDT.

GDT did not halt or reverse the course of early-stage AKI. However, GDT partially reversed global hypoperfusion and resulted in decreased in-hospital mortality.

## Figures and Tables

**Table 1:** Baseline demographic and clinical characteristics of the patients in the control and GDT groups.

	Control n=51	GDT n=48	*p* value
Age, years	66.7±13.5	64.6±14.3	0.467
Male (%)	33 (64.7)	29 (60.4)	0.656
Body weight	69.4±14.8	66.5±14.8	0.337
APACHE II score	15.5 [11.8-22.0]	16.0 [10.0-19.0]	0.351
SOFA score	4.5 [2.0-7.0]	4.0 [2.0-7.5]	0.797
Baseline creatinine	1.21 [1.10-1.72]	1.36 [1.10-1.72]	0.13
Neoplasia	28 (54.9%)	27 (56.3%)	0.893
Diabetes mellitus	11 (21.6)	5 (10.4)	0.132
Autoimmune disease	3 (5.9)	1 (2.1)	0.618
Use of nephrotoxic drugs	7 (13.7)	8 (16.7)	0.683
Septic shock	14 (27.5)	18 (37.5)	0.285
Source (%)			0.438
Medical	8 (15.7)	5 (10.4)	
Surgical	43 (84.6)	43 (89.6)	
Urgency	16 (31.4)	16 (33.3)	0.740
Type of Surgery (%)			0.799
Gastrointestinal	29 (67.4)	29 (69.0)	
Orthopedic	6 (14)	3 (7.1)	
Vascular	2 (4.7)	1 (2.4)	
Thoracic	3 (7.0)	3 (7.1)	
Neurosurgery	2 (4.7)	3 (7.1)	
Others	1 (2.3)	3 (7.1)	

Numbers are presented as n (%), the mean ± standard deviation, or as the median [IQR].

**Table 2:** Interventions during the 8-h treatment period in the control and GDT groups.

Interventions	Control n=51	GDT n=48	*p* value
Fluid challenge (%)	29 (56.9)	44 (91.7)	<0.001
Fluid challenges per patient	2 [1.0-3.0]	3 [1.8-4.2]	0.008
Fluid challenge with crystalloids (%)	17 (33.0)	44 (91.7)	<0.001
Crystalloids, ml	1230±906	1750±1053	0.318
Fluid challenge with colloids (%)	12 (23.5)	0 (0)	-
Colloids, mL	750±412	0 (0)	-
Transfusion (%)	2 (3.9)	26 (54.2)	<0.001
Red blood cells, units	2±1.4	1.6±0.8	0.517
Dobutamine treatment (%)	4 (7.8)	38 (79.2)	0.001
Dobutamine dose, μg/kg.min	3.3±1.0	4.0±1.9	0.023
Noradrenalin treatment (%)	17 (33)	32 (67)	0.001
Noradrenalin dose, μg/kg.min	0.34±0.41	0.31±0.39	0.710
Sodium nitroprusside treatment (%)	1 (2.0)	6 (12.0)	0.054
Furosemide treatment (%)	8 (15.7)	1 (2.1)	0.031

Numbers are presented as n (%), the mean±standard deviation, or as the median [IQR].

**Table 3:** Vital signs, central venous pressure, and oxygenation and perfusion variables during the 8-h treatment period in the control and GDT groups.

	Group	0h	2h	4h	6h	8h
HR (bpm)	Control	99 [82-117]	101 [79.5-113.5]	106 [82.5-118]	102 [84-116.5]	108 [83-126]*
	GDT	91 [76-107]	98 [86-109]	104 [83-116]*	104 [89-113]*	101 [89-113]*
MAP (mmHg)	Control	80 [75-89]	82 [71-93]	78 [70-88]	80 [71-96]	81 [72-96]
	GDT	80 [73-90]	82 [73-96]	77 [70-90]	79 [70-90]	77 [68-89]
CVP (mmHg)	Control	12 [8-15]	13 [8-17]	12 [9-17]	13 [9-17]	13 [10-17]
	GDT	9 [6-13]^#^	10 [8-15]*	9 [7-14]	10 [8-13]^#^	10 [8-15]*
SaO2 (%)	Control	96 [94-97]	96 [94-97]	95 [93-97]	96 [94-97]	96 [94-98]
	GDT	96 [93-97]	96 [95-97]	96 [94-97]	96 [94-97]	96 [94-98]
Hb (mg/dL)	Control	10.0 [9.3-11.0]	-	9.9 [8.8-11.1]	-	9.7 [8.4-10.5]
	GDT	9.8 [8.8-11.0]	-	10.0 [9.1-10.8]	-	9.9 [9.2-10.6]
ScvO2 (%)	Control	74 [65-78]	-	73 [55-80]	-	75 [65-83]
	GDT	74 [68-80]	-	77 [71-82]*^#^	-	79 [75-81]*^#^
Lactate (mEq/L)	Control	1.8 [1.4-3.0]	-	1.9 [1.6-3.2]	-	1.6 [1.3-2.5]
	GDT	1.8 [1.3-3.4]	-	1.6 [1.1-2.4]*	-	1.3 [1-2.0]*

Numbers are presented as the median [IQR]. *: *p*<0.05 *vs*. baseline. ^#^: *p*<0.00125 *vs*. the control group.

**Table 4:** Outcomes.

Outcomes	Control n=51	GDT n=48	*p*-value
**Primary outcomes**			
Scr difference 8h	0.10 [0.00-0.37]	0.10 [-0.10-0.40]	0.482
Scr difference day 1	0.00 [-0.10-0.30]	0.10 [-0.20-0.60]	0.960
Scr difference day 2	0.10 [-0.14-0.60]	0.17 [-0.25-0.80]	0.768
Scr difference day 3	0.10 [-0.30-0.54]	0.10 [-0.35-0.70]	0.914
RRT, n (%)	12 (25.0)	13 (27.1)	0.818
8-h diuresis, ml	396.5 [200.0-500.0]	397.5 [252.5-537.5]	0.515
AKI beyond 72h, n (%)	40 (78.4)	30 (62.5)	0.064
**Secondary outcomes**			
ICU LOS, days	7.5 [4.0-14.0]	7.0 [5.0-13.0]	0.579
Hospital LOS, days	15.0 [10.0-25.5]	18.5 [10.0-27.0]	0.745
ICU mortality, n (%)	15 (29.4)	10 (20.8)	0.326
Hospital mortality, n (%)	26 (51.0)	15 (31.3)	0.048
Composite, n (%)	44 (86.3)	37 (77.1)	0.178

SCr difference: Difference in serum creatinine level (8h and 1, 2, and 3 days after minus before initiation of goal-directed therapy). Composite: composite of hospital mortality, renal replacement therapy, or persistent renal functional impairment after 72h. Numbers are presented as n (%) or the median [IQR].
